# Isolated proteinuria due to *CUBN* homozygous mutation – challenging the investigative paradigm

**DOI:** 10.1186/s12882-019-1474-z

**Published:** 2019-08-22

**Authors:** Kushani Jayasinghe, Susan M. White, Peter G. Kerr, Duncan MacGregor, Zornitza Stark, Ella Wilkins, Cas Simons, Andrew Mallett, Catherine Quinlan

**Affiliations:** 1 Department of Nephrology, Monash Medical Centre, Melbourne, Australia; 2 Monash University, Melbourne, Australia; 3 Murdoch Children’s Research Institute, Melbourne, Australia; 4 The KidGen Collaborative, Australian Genomics Health Alliance, Victoria, Australia; 5 Department of Paediatrics, University of Melbourne, Victoria, Australia; 6 Victorian Clinical Genetics Services, Murdoch Children’s Research Institute, Melbourne, Australia; 7 Department of Pathology, Royal Children’s Hospital, Melbourne, Australia; 8 Kidney Health Service and Conjoint Renal Research Laboratory, Royal Brisbane and Women’s Hospital, Brisbane, Australia; 9 Institute for Molecular Bioscience and Faculty of Medicine, The University of Queensland, Brisbane, Australia; 10 Department of Paediatric Nephrology, Royal Children’s Hospital, 50 Flemington Street, Parkville, Australia

**Keywords:** Genomics, Genetics, Chronic kidney disease

## Abstract

**Background:**

Proteinuria is a common clinical presentation, the diagnostic workup for which involves many non-invasive and invasive investigations. We report on two siblings that highlight the clinically relevant functional role of cubulin for albumin resorption in the proximal tubule and supports the use of genomic sequencing early in the diagnostic work up of patients who present with proteinuria.

**Case presentation:**

An 8-year-old boy was referred with an incidental finding of proteinuria. All preliminary investigations were unremarkable. Further assessment revealed consanguineous family history and a brother with isolated proteinuria. Renal biopsy demonstrated normal light microscopy and global glomerular basement membrane thinning on electron microscopy. Chromosomal microarray revealed long continuous stretches of homozygosity (LCSH) representing ~ 4.5% of the genome. Shared regions of LCSH between the brothers were identified and their further research genomic analysis implicated a homozygous stop-gain variant in *CUBN* (10p12.31).

**Conclusions:**

*CUBN* mutations have been implicated as a hereditary cause of megaloblastic anaemia and variable proteinuria. This is the second reported family with isolated proteinuria due to biallelic *CUBN* variants in the absence of megaloblastic anaemia, demonstrating the ability of genomic testing to identify genetic causes of nephropathy within expanding associated phenotypic spectra. Genomic sequencing, undertaken earlier in the diagnostic trajectory, may reduce the need for invasive investigations and the time to definitive diagnosis for patients and families.

## Background

Proteinuria is a common clinical presentation that is associated with poor renal outcomes, especially when severe [1]. Historically, the diagnostic workup of patients with isolated proteinuria involved urinalysis, imaging and blood sampling before potentially proceeding to histological diagnosis via renal biopsy. Many nephropathies can cause proteinuria, which gives rise to indistinguishable phenocopies, even when histological examination is performed. This is particularly the case in children. Recently, advances in technology has resulted in the reduced cost and increased availability of genomic sequencing for establishing a clinical diagnosis [2]. In addition, preliminary studies have demonstrated genomics to be useful as a diagnostic test in selected patients with kidney disease [3]. Although current treatment strategies target glomerular filtration of albumin, more recent data suggests there are also clinically relevant functions of the proximal tubule in albumin homeostasis. We present the cases of two siblings which supports the clinically relevant functional role of cubulin for albumin resorption in the proximal tubule and suggest that genomic sequencing be undertaken early in the diagnostic work up of patients presenting with proteinuria.

## Case presentation

**Fig. 1:**
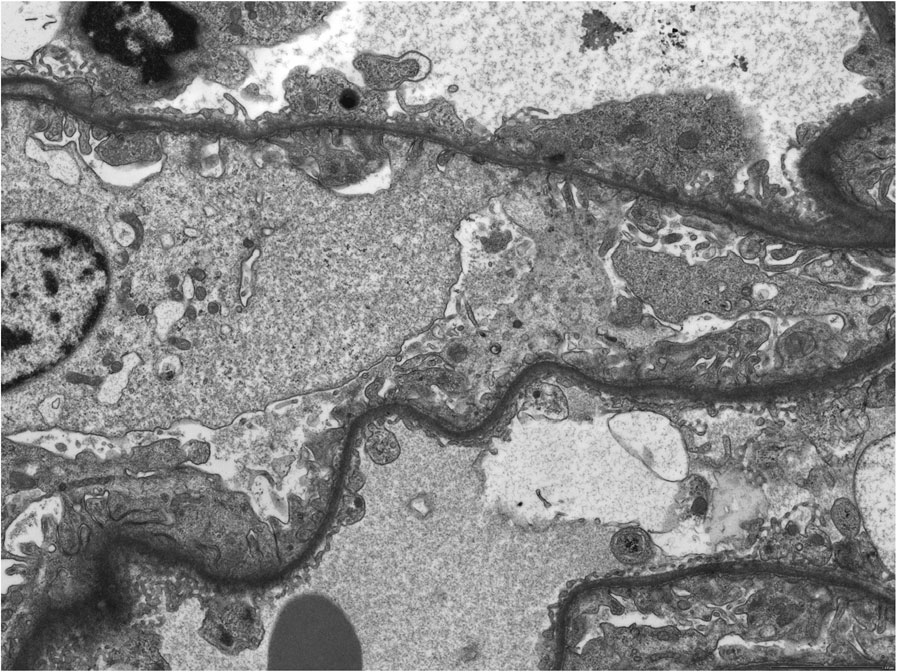
Electron Microscopy demonstrating global uniform thinning of glomerular basement membrane

An 8-year-old boy was referred to the paediatric nephrology service with an incidental finding of persistent proteinuria during investigation for abdominal pain. He had no hematuria or glycosuria. He had normal serum albumin (42 g/L). Haematological, biochemical and immunological parameters were unremarkable. Specifically, C3, C4, ANCA and anti-GBM were negative and ANA was moderately positive (1:320). Renal function was normal (serum creatinine 0.52 mg/dL). Urinary albumin creatinine ratio was raised at 51.4 mg/mmol (normal < 3.5/mg/mmol) and protein creatinine ratio 92 mg/mmol (normal< 20 mg/mmol). Urine electrophoresis was 1.47 g/L with non-selective proteinuria pattern detected (normal< 0.10 g/L). Assessment for glycosuria, urinary light chains and urinary amino acids was negative. Urinary electrolytes were normal, and haematuria was absent on serial testing. Renal ultrasound, ophthalmology and audiology assessment did not demonstrate any abnormal findings. Renal biopsy demonstrated normal light microscopy and there was no evidence of immune complex mediated disease or other pathology. Podocyte foot processes were generally well preserved on electron microscopy, however there was global uniform thinning of the glomerular basement membrane (Fig. 1).

Family history assessment revealed that his parents were first cousins, of Middle Eastern descent, with no known family history of renal disease or other comorbidity. Further investigation of the family revealed isolated proteinuria (urinary albumin creatinine 55.6 mg/mmol, protein creatinine 89.4 mg/mmol) in his 4-year old brother but not in either parent. His brother’s haematological parameters were unremarkable and remaining urinary investigations were within normal range apart from an elevated urinary protein electrophoresis (1.32 g/L). Chromosomal microarray in the older sibling revealed long continuous stretches of homozygosity (LCSH) representing ~ 4.5% of the genome, consistent with parental consanguinity. Shared regions of LCSH between the brothers were interrogated using the Genomic Oligoarray and single nucleotide polymorphism (SNP) evaluation tool© [4]. Whole genome sequencing was performed on both affected individuals after informed consent (HREC/15/QRCH/126). Segregating variants were examined in the previously identified areas of LCSH and in all genomic areas in a curated list of glomerular disease associated genes [5]. This revealed both brothers had a homozygous stop-gain variant NM_001081.3:c.4689_4690delTAinsAT; p.(Cys1563Ter) in exon 31 of the gene *CUBN*. Biallelic variants in *CUBN* have previously been associated with Imerslund-Grasbeck Syndrome (Megaloblastic anemia-1, Finnish type (MIM: 261100)) and one reported case of isolated proteinuria [6–8]. The c.4689_4690delTAinsAT variant is present in 47 individuals in the gnomAD database including a single homozygous individual. The variant is most common in the “South Asian” gnomAD population with an allele frequency of 0.0012. Based on this evidence the variant was classified as pathogenic according to ACMG criteria (PVS1, PM2 and PP1) [9]. At four years of follow-up, the patient and his younger brother remain well with isolated proteinuria. Given the genomic diagnosis, biopsy of the younger brother was not required. Both were commenced on renin-angiotensin-aldosterone system (RAAS) blockade with a mild reduction in proteinuria (urine protein:creatinine 74.6 mg/mmol in the elder brother and 73.8 mg/mmol in the younger brother). Haematological parameters remained normal in both siblings.

## Discussion and conclusions

The causes of isolated proteinuria can be divided into glomerular, tubular and overflow proteinuria. Glomerular proteinuria occurs in nephrotic syndrome due to increased permeability of the glomerular capillary wall, resulting in the abnormal passage of proteins from the glomerulus [10]. Tubular proteinuria which occurred in this case, is typically due to increased excretion of low molecular weight proteins, such as beta-2 microglobulin, retinol binding protein and vitamin D-binding protein (DBP) due to abnormalities in proximal tubular reabsorption of these proteins [11]. A classic example is Fanconi syndrome, which has acquired and inherited associations. Lastly, overflow proteinuria is due to overproduction of an individual protein, for example in multiple myeloma. We infer that the *CUBN* mutation in our patients may have led to predominant albuminuria due to a deficiency in tubular reabsorption of albumin.

The role of megalin and cubulin in proximal proteinuria has been postulated in several studies, with the most recent study demonstrating that while megalin/cubulin mouse knockouts result in blocked proximal tubular albumin uptake, this did not affect plasma albumin levels in nephrotic mice [12] . This suggests there is not a significant contribution of the megalin/cubulin pathway for albumin homeostasis. A prior study nevertheless reported a 20% decrease in plasma albumin levels in *cubn* heterozygous mice [13]. Future studies are required to clarify the role of the proximal tubule in albumin homeostasis.

*CUBN* mutations have previously been described as causative in Imerslund-Grasbeck Syndrome [14], with over 20 families have been reported with megaloblastic anemia and proteinuria associated with truncating bi-allelic *CUBN* mutations [6, 7]. Our family is the second described in the literature as having presented with *isolated* proteinuria as their only phenotypic feature. The previous family was described by Ovunc et al as having a novel biallelic 1-bp homozygous deletion in *CUBN* resulting in a phenotype of fluctuating proteinuria in otherwise well individuals [8]. Of note, Boger et al identified a missense variant (p.I2984V) in *CUBN* associated with albuminuria in a genome wide association study [15]. Further, an association between a *CUBN* single-nucleotide polymorphisms (SNP) (rs1801239) and ESKD has been demonstrated in African Americans [16].

Persistent proteinuria is an important marker of chronic kidney disease and associated with inferior cardiovascular and renal outcomes [1], and serial dipstick proteinuria has been identified as an effective strategy to determine those at risk of rapid renal decline [17]. Most studies do not differentiate between glomerular or tubular proteinuria, however heavy proteinuria has been associated with adverse outcomes in the absence of complete surety about its glomerular versus tubular source. Isolated 2+ or 100 mg per dL proteinuria on dipstick is associated with a greater risk of end stage kidney disease than an eGFR < 60 ml/min/1.73 m2 in a 25-year follow up study of men without identifiable kidney disease [18]. Current diagnostic workup for proteinuria involves a careful history and examination, multiple urine samples for confirmation and quantification of proteinuria and baseline haematology and biochemistry. This is then followed by renal ultrasound and other increasingly complex laboratory tests for specific causes of glomerulonephritis or glomerular pathology if indicated [19]. The role of renal biopsy in the investigation of asymptomatic isolated albuminuria is controversial, particularly in paediatrics, where percutaneous renal biopsy has a reported major complication rate of up to 30% [20–22].

Currently treatment strategies are focussed on glomerular proteinuria, largely by reducing glomerular filtration pressure with renin-angiotensin system inhibition and blood pressure control. Although this has been shown to be effective in reducing decline in kidney function [23], future directions for therapy should explore targets beyond glomerular filtration, including the proximal tubule. Previously, the proximal tubule was thought to be non-permeable to albumin. More recent data challenges this concept suggesting an identifiable role for the proximal tubule in reabsorption of filtered albumin [24]. The megalin-cubulin complex is one identified mechanism for proximal tubular uptake of albumin that is filtered through the glomerular filtration barrier. The role of this complex has been investigated in knockout animal models [12]. There are, however, some differences between human and mouse kidney models. Increase in urinary DBP was observed in megalin-deficient and cubulin-deficient humans [7, 25], but not in cubulin-deficient mice [26] that had intact megalin. This indicates that functional megalin is sufficient for normal tubular reabsorption in mice, but not in humans.

This family, in addition to the one previously described in the literature provides further evidence for gene-disease validity association, which will be important in guiding the interrogation and interpretation of genomic data in renal patients presenting with proteinuria [27]. We propose that isolated proteinuria could result from mutations in *CUBN* secondary to deficiency of albumin uptake by the proximal tubule, resulting in a renal limited form of Imerslund-Grasbeck Syndrome. This may represent a phenocopy of other isolated proteinuric kidney diseases. Although the patient in our case also demonstrated thinning of the GBM on biopsy, we do not believe that this was the cause of his proteinuria due to the absence of haematuria and other structural anomalies on light and electron microscopy. This family highlights the need to include *CUBN* in the genomic diagnostic approach to proteinuria. As the availability of next-generation-sequencing (NGS) increases and costs decrease, genomic testing is now being increasingly considered a part of the standard workup of chronic kidney disease. We suggest genomic testing should be performed in patients with proteinuria with a strong suspicion of genetic disease, antecedent to or concurrent with renal biopsy depending upon clinical scenario and urgency, particularly where markers of proximal tubular proteinuria are present [28].

*CUBN* mutations have been implicated as a hereditary cause of megaloblastic anaemia and variable proteinuria. This family is the second described in the literature to have a monogenic *CUBN-*mediated cause of isolated proteinuria, approximating a renal-limited form of Imerslund-Brasbeck Syndrome and phenocopying other forms of isolated proteinuria. Therefore, we describe how genomic sequencing can successfully identify a single gene cause of nephropathy in a clinically relevant and applicable manner. This report strengthens the gene-disease validity association for *CUBN*, with implications for the practice of diagnostic laboratories. Furthermore, this case supports the argument that genomic sequencing should be undertaken earlier in the diagnostic workup in order to reduce the need for invasive investigations, such as renal biopsy. Future cases with long term follow up of isolated proteinuria due to *CUBN* mutations will help to inform the management and prognosis of these patients.

## Data Availability

The datasets used and/or analysed during the current study are available from the corresponding author on reasonable request.

## Abbreviations

ANA: Anti-nuclear antibody
ANCA: Anti-neutrophil cytoplasmic antibody
anti-GBM: Anti-glomerular basement membrane
C3: Complement component 3
C4: Complement component 4
HREC: Human research ethics committee DBP Vitamin D binding protein
LCSH: Long continuous stretches of homozygosity
NGS: Next-generation-sequencing
SNP: Single-nucleotide polymorphisms

## References

[1] Hemmelgarn BR, Manns BJ, Lloyd A, James MT, Klarenbach S, Quinn RR (2010). Relation between kidney function, proteinuria, and adverse outcomes. Jama..

[2] Rabbani B, Mahdieh N, Hosomichi K, Nakaoka H, Inoue I (2012). Next-generation sequencing: impact of exome sequencing in characterizing Mendelian disorders. J Hum Genet.

[3] Lata S, Marasa M, Li Y, Fasel DA, Groopman E, Jobanputra V (2018). Whole-exome sequencing in adults with chronic kidney disease: a pilot study. Ann Intern Med.

[4] Wierenga Klaas J., Jiang Zhijie, Yang Amy C., Mulvihill John J., Tsinoremas Nicholas F. (2012). A clinical evaluation tool for SNP arrays, especially for autosomal recessive conditions in offspring of consanguineous parents. Genetics in Medicine.

[5] Little MH, Quinlan C. Advances in our understanding of genetic kidney disease using kidney organoids. Pediatr Nephrol. 2019. 10.1007/s00467-019-04259-x. [Epub ahead of print]. 10.1007/s00467-019-04259-x 31065797

[6] Grasbeck R (2006). Imerslund-Grasbeck syndrome (selective vitamin B(12) malabsorption with proteinuria). Orphanet J Rare Dis.

[7] Storm T, Emma F, Verroust PJ, Hertz JM, Nielsen R, Christensen EI (2011). A patient with Cubilin deficiency. N Engl J Med.

[8] Ovunc B, Otto EA, Vega-Warner V, Saisawat P, Ashraf S, Ramaswami G (2011). Exome sequencing reveals cubilin mutation as a single-gene cause of proteinuria. J Am Soc Nephrol.

[9] Rehm HL, Bale SJ, Bayrak-Toydemir P, Berg JS, Brown KK, Deignan JL (2013). ACMG clinical laboratory standards for next-generation sequencing. Genet Med.

[10] Michael AF, McLean RH, Roy LP, Westberg NG, Hoyer JR, Fish AJ (1973). Immunologic aspects of the nephrotic syndrome. Kidney Int.

[11] Rabelink TJ, Heerspink HJL, de Zeeuw D, Kimmel PL, Rosenberg ME (2015). Chapter 9 - the pathophysiology of proteinuria. Chronic renal disease.

[12] Weyer K, Andersen PK, Schmidt K, Mollet G, Antignac C, Birn H (2018). Abolishment of proximal tubule albumin endocytosis does not affect plasma albumin during nephrotic syndrome in mice. Kidney Int.

[13] Aseem O, Smith BT, Cooley MA, Wilkerson BA, Argraves KM, Remaley AT (2014). Cubilin maintains blood levels of HDL and albumin. J Am Soc Nephrol.

[14] Aminoff M, Carter JE, Chadwick RB, Johnson C, Grasbeck R, Abdelaal MA (1999). Mutations in CUBN, encoding the intrinsic factor-vitamin B12 receptor, cubilin, cause hereditary megaloblastic anaemia 1. Nat Genet.

[15] Boger CA, Chen MH, Tin A, Olden M, Kottgen A, de Boer IH (2011). CUBN is a gene locus for albuminuria. J Am Soc Nephrol.

[16] Ma J, Guan M, Bowden DW, Ng MC, Hicks PJ, Lea JP (2016). Association analysis of the Cubilin (CUBN) and Megalin (LRP2) genes with ESRD in African Americans. Clin J Am Soc Nephrol.

[17] Clark WF, Macnab JJ, Sontrop JM, Jain AK, Moist L, Salvadori M (2011). Dipstick proteinuria as a screening strategy to identify rapid renal decline. J Am Soc Nephrol.

[18] Ishani A, Grandits GA, Grimm RH, Svendsen KH, Collins AJ, Prineas RJ (2006). Association of single measurements of dipstick proteinuria, estimated glomerular filtration rate, and hematocrit with 25-year incidence of end-stage renal disease in the multiple risk factor intervention trial. J Am Soc Nephrol.

[19] Lunn A, Forbes TA (2016). Haematuria and proteinuria in childhood. Paediatr Child Health.

[20] Simckes A. M., Alon Uri S., Blowey Douglas L., Gyves Katherine M. (2000). Success and safety of same-day kidney biopsy in children and adolescents. Pediatric Nephrology.

[21] Feneberg R, Schaefer F, Zieger B, Waldherr R, Mehls O, Scharer K (1998). Percutaneous renal biopsy in children: a 27-year experience. Nephron..

[22] Sinha MD, Lewis MA, Bradbury MG, Webb NJ (2006). Percutaneous real-time ultrasound-guided renal biopsy by automated biopsy gun in children: safety and complications. J Nephrol.

[23] Wuhl E, Trivelli A, Picca S, Litwin M, Peco-Antic A, Zurowska A (2009). Strict blood-pressure control and progression of renal failure in children. N Engl J Med.

[24] Dickson LE, Wagner MC, Sandoval RM, Molitoris BA (2014). The proximal tubule and albuminuria: really!. J Am Soc Nephrol.

[25] Storm T, Tranebjaerg L, Frykholm C, Birn H, Verroust PJ, Neveus T (2013). Renal phenotypic investigations of megalin-deficient patients: novel insights into tubular proteinuria and albumin filtration. Nephrol Dial Transplant.

[26] Amsellem S, Gburek J, Hamard G, Nielsen R, Willnow TE, Devuyst O (2010). Cubilin is essential for albumin reabsorption in the renal proximal tubule. J Am Soc Nephrol.

[27] Strande NT, Riggs ER, Buchanan AH, Ceyhan-Birsoy O, DiStefano M, Dwight SS (2017). Evaluating the clinical validity of gene-disease associations: an evidence-based framework developed by the clinical genome resource. Am J Hum Genet.

[28] Monnens L, Levtchenko E (2008). Evaluation of the proximal tubular function in hereditary renal Fanconi syndrome. Nephrol Dial Transplant.

[29] Posters. Nephrology. 2018;23(S3):71–100.

